# The spatial-temporal clustering of *Plasmodium falciparum *infection over eleven years in Gezira State, The Sudan

**DOI:** 10.1186/1475-2875-9-172

**Published:** 2010-06-18

**Authors:** Samia E Mirghani, Bakri YM Nour, Sayed M Bushra, Ibrahim M Elhassan, Robert W Snow, Abdisalan M Noor

**Affiliations:** 1 University of Gezira, Blue Nile National Institute for Communicable Diseases, P.O. Box 101, Wad-Medani, Sudan; 2 Institute of Endemic Diseases, Department of Parasitology, University of Khartoum, Sudan; 3 Faculty of Medicine, University of Jazan, Kingdom of Saudi Arabia; 4 Malaria Public Health and Epidemiology Group, Centre for Geographic Medicine, KEMRI - University of Oxford - Wellcome Trust Collaborative Programme, Kenyatta National Hospital Grounds, P.O. Box 43640-00100, Nairobi, Kenya; 5 Centre for Tropical Medicine, Nuffield Department of Clinical Medicine, University of Oxford, CCVTM, Oxford OX3 7LJ, UK

## Abstract

**Background:**

Malaria infection and disease exhibit microgeographic heterogeneity which if predictable could have implications for designing small-area intervention. Here, the space-time clustering of *Plasmodium falciparum *infections using data from repeat cross-sectional surveys in Gezira State, a low transmission area in northern Sudan, is investigated.

**Methods:**

Data from cross-sectional surveys undertaken in January each year from 1999-2009 in 88 villages in the Gezira state were assembled. During each survey, about a 100 children between the ages two to ten years were sampled to examine the presence of *P. falciparum *parasites. In 2009, all the villages were mapped using global positioning systems. Cluster level data were analysed for spatial-only and space-time clustering using the Bernoulli model and the significance of clusters were tested using the Kulldorff scan statistic.

**Results:**

Over the study period, 96,022 malaria slide examinations were undertaken and the *P. falciparum *prevalence was 8.6% in 1999 and by 2009 this had reduced to 1.6%. The cluster analysis showed the presence of one significant spatial-only cluster in each survey year and one significant space-time cluster over the whole study period. The primary spatial-only clusters in 10/11 years were either contained within or overlapped with the primary space-time cluster.

**Conclusion:**

The results of the study confirm the generally low malaria transmission in the state of Gezira and the presence of spatial and space-time clusters concentrated around a specific area in the south of the state. Improved surveillance data that allows for the analysis of seasonality, age and other risk factors need to be collected to design effective small area interventions as Gezira state targets malaria elimination.

## Background

Malaria parasite transmission and clinical disease are characterized by important microgeographic variations, often between adjacent villages, households or families [1-7]. This local heterogeneity is driven by a variety of factors including genetic [6,8], distance to potential breeding sites [9-12], housing construction [2,5,11,13,14], presence of domestic animals near the household [15,16], and socio-behavioural characteristics [3,10,11,17]. While seldom prioritized in the planning of malaria control by national programmes, the understanding of the microepidemiology of malaria is important to the design of effective small-area interventions [3,4] particularly in areas of unstable or very low transmission where risk is over-dispersed and highly focal [18].

To assess space-time local heterogeneity of disease, techniques that detect the presence of statistically significant small-area disease clusters are often used [5,7,19-21]. Some of the earliest use of disease cluster analysis was in the detection of distribution patterns of rare conditions such as cancers [20,22] and more recently applied to infectious diseases including dengue [23], filariasis [24], sleeping sickness [25] and West Nile virus [26]. The space-time clustering of malaria has also been described but mainly in moderate to high transmission settings [2,5,27-30]. There are very few descriptions and quantification of space-time clustering of infection, disease or hospitalization from malaria in areas of unstable low or very low transmission settings. Here, the spatial and temporal clustering of *Plasmodium falciparum *infections in 88 villages surveyed each year from 1999 to 2009 in the Gezira state, a generally very low unstable transmission area of the northern Sudan, is examined.

## Methods

### Study area

**Figure 1:**
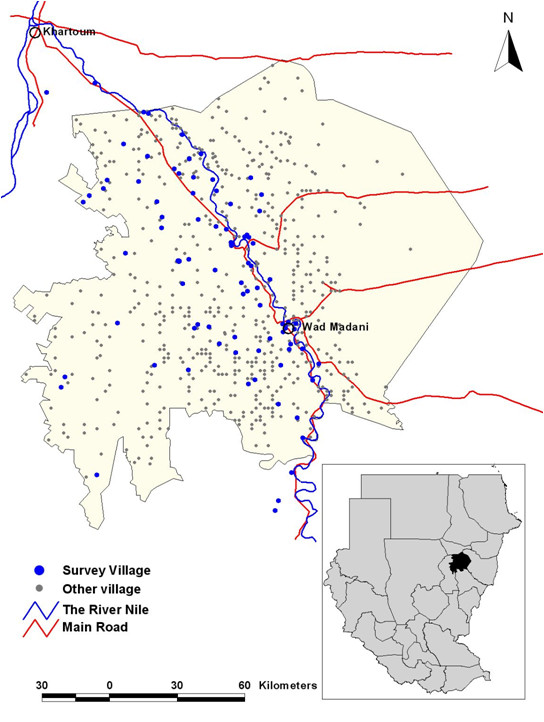
**Map of Gezira state showing the location of the state capital (Wad Madani) in relation to the national capital (Khartoum), the distribution of settlements in Gezira and the location of the distribution of 88 survey locations where the *P. falciparum *prevalence surveys were undertaken from 1999-2009**. Inset is the state map of the Sudan showing the location of Gezira state.

Gezira State is in the eastern central region of Sudan and has an area of 23,373 km² and an estimated population of approximately 3.2 million people according to the 2008 national census [CBS 2008 unpublished data]. The State is dissected by the Blue Nile River with Wad Madani town as its capital (Figure 1). The average daily temperature is 32°C during summer (April, May, June to middle of July) and 22°C during winter (November to the end of January). The rainy season starts in July and ends by October, with an estimated annual rainfall of 140 - 225 mm [31]. The relative humidity is 38% in autumn (end of July to the middle of October) and 30% during winter. The Gezira irrigation scheme is the main economic activity in the state and a major challenge for malaria control as it contributes to the accumulation of water resulting in both permanent and temporary breeding sites for mosquitoes and other vectors that cause water-borne diseases. *Anopheles arabiensis *is the main vector of malaria throughout in Gezira state [31,32]. The peak months of malaria transmission are from September to November. In the irrigated areas, however, a third peak of malaria transmission in February to March is often observed as a result of increased mosquito densities coinciding with the end of the irrigation season, when there are many pools of stagnant water along the drying rivers and canals [32].

### Malaria control in Gezira state

Malaria control in Gezira evolved to address the increasing risk of infection due to the establishment of the Gezira irrigation scheme in 1925 with vector control as the primary approach [31]. From 1935 when formal malaria control began, Gezira state has seen periods of dramatic success when *P. falciparum *prevalence dropped to below 1% in 1970 punctuated by resurgence of infections leading to epidemics in 1971, 1974, 1993 and 1994 [31,33]. These epidemics have been attributed to the interruption of malaria control due to lack of funding [31,33] and the emergence of resistance to insecticides after sustained periods of use [31,34,35]. In 1975, the Gezira State Malaria Control Programme (GMCP) of the Federal Ministry of Health (FMoH) began longitudinal surveys of *P. falciparum *prevalence among children aged between 2 to below 10 years to monitor disease patterns in the state [33]. In 1978, the Blue Nile Health Project was established to ensure long-term malaria control in the area around the Gezira irrigation scheme supported by carefully assembled scientific evidence [31]. This was followed by sustained malaria control in the project area for 10 years before funding was withdrawn in 1989 resulting in a resurgence of malaria incidence [33].

Since 1999, the GMCP has implemented a number of malaria control initiatives consisting mainly of indoor residual spraying (IRS) of households and larviciding of breeding sites in all localities [33]. In 2002, the Gezira State Malaria Free Initiative (GMFI) was created with support from the WHO and the FMoH [33]. In the same year, insecticide-treated nets were introduced and are now provided to households in all localities. From 1999 to 2001 the two main insecticides for IRS were pyrethroid combinations, permethrin and malathion-deltamethrin, while temephos (Abate) EC 50% was the main insecticide for larviciding throughout the study period [33]. In 2002, following reports of emerging resistance to malathion-deltamethrin this insecticide was discontinued and only permethrin was used for IRS, augmented with use of pyrethroid-treated bed nets. Permethrin was replaced with bendiocarb for IRS in 2007 and 2008 when reduced susceptibility of vectors to pyrethroids was reported in 2005 and 2006 [36]. In 2009, IRS was not implemented, but larviciding using temephos EC 50% and the distribution of long-lasting insecticide-treated nets continued across the whole state (Sayed El Bushra, Personal Communication).

### Parasitological surveys

A random sample of 88 villages covering all localities of Gezira state was selected and in each village children between the ages of two to below ten years were examined for malaria parasites [33]. These surveys were undertaken by field teams from the GMCP in January each year. Originally they covered 154 villages but only the 88 villages were used in this study (Figure 1), as they represented the most contemporary and temporally complete dataset within the entire data series covering the period 1999-2009. During the surveys, the team leaders explained the survey objectives to the household head before blood samples were drawn from the selected children of consenting parents/guardians. In every village, all children two to below ten years were listed and a 100 were randomly selected. For all children, thick and thin blood smears for malaria parasites were prepared and allowed to dry. Once dry, they were stored in slides boxes and transported to a central laboratory at the GMCP in Wad Madani town. The thin smears were fixed in absolute methanol. The thick and thin smears were then stained in 4% Giemsa solution for 30 minutes and were subsequently read using a light microscope with a ×100 oil-immersion lens and ×10 eyepieces. One hundred high power fields were examined before a slide was considered negative for the presence of parasites. After the first reading of all slides, all positive and a randomly selected proportion of the negative slides were read by an independent microscopist for quality control. All children who were found positive for the malaria parasites in the first reading were visited at their home by the state malaria team and were treated with the first-line drug, which was chloroquine (CQ) or sulphadoxine-pyrimethamine (SP) prior to 2004 and artesunate-SP (AS+SP) since 2004. In 2009, all the villages were mapped using Garmin *etrex *(Garmin Inc., Kansas, USA) handheld global positioning systems (GPS). The final database of villages contained information on the name of the village, the longitude and latitude, the year of survey, and a summary of the number of children who were examined and the number who were positive for *P. falciparum *parasites. Individual level data were not available for analysis.

### Spatial and temporal cluster analysis

The Kulldorff spatial scan statistic [20], as implemented in SaTScan 8.0 [37] was used for the analysis of the spatial and temporal clustering of the data, with the specific aim of identifying clusters of high *P. falciparum *infection rates. A Bernoulli model was used for the analysis of spatial and temporal clustering in the data for several reasons. First, the number of people surveyed in some locations varied over the years and it was important to adjust for these sampling changes to avoid clusters that are driven by the number of people surveyed rather than the number of people who had infection. Second, the model allowed for locations or years that are always of high malaria prevalence relative to other locations/years to contribute to the overall space-time clusters, a particularly important advantage given the generally low prevalence of the survey locations throughout the study period. Third, this model allowed analysis of the purely spatial and/or the space-time scan statistics [19,20]. The Bernoulli model requires the case and control data, represented respectively by *P. falciparum *positive and negative samples, and the spatial location and time for each case and control [19,20]. A circular spatial scan window and a maximum spatial cluster size of 50% of the cases was used so that both small and large clusters could be detected. The model compares the number of observed cases and controls in a cluster to the expectation if the spatial and temporal locations of all cases were assumed to be independent of each other so that there is no space-time interaction. Tests of statistical significance of the identified clusters were based on likelihood ratio tests, with P-values obtained by 9999 Monte Carlo replications. The main model outputs were the location and radius of clusters, the number of villages in the cluster, the number of observed and expected cases, the rate ratio defined as the ratio of observed to expected cases of *P. falciparum *infection and the P-value of the Kulldorff scan statistic.

### Ethical approval

Ethical approval for this study was obtained from the Ethical Committee of the Blue Nile Research National Institute for Communicable Diseases - University of Gezira. Formal permission was obtained from the Gezira malaria control programme of the State Ministry of Health. Consent was sought from parents/guardians before blood samples were collected from sampled children.

## Results

### *Plasmodium falciparum *infection prevalence

**Figure 2:**
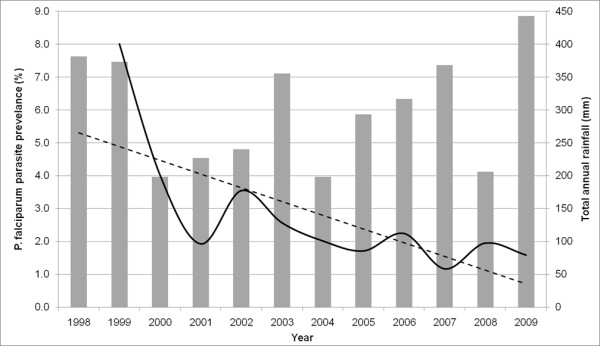
**Graph of *P. falciparum *prevalence (solid line) and the total rainfall of the preceding year in mm (bars) as estimated from meteorological stations in Gezira state by year of survey**. The dashed line represents the linear trend line (R^2 ^= 0.522) of *P. falciparum *prevalence from 1999-2009.

Over the period 1999 to 2009, 96,022 malaria slide examinations were undertaken among children aged between two to below ten years in 88 villages in Gezira state (Table 1). There were no major differences in total observations between years with a mean annual number of observations of 8,729 (8,523 - 8,928) children. At the start of the study period in 1999, 8.6% of the children examined were positive for *P. falciparum *infection and by 2009 this had reduced to 1.6% representing more than a five-fold decline in overall *P. falciparum *infection rates (Table 1 and Figure 2, R^2 ^of the trendline = 0.52). Although throughout the survey period there was a consistent decline in infection rates with time, the trend also showed a number of perceptible peaks in infection in 2002, 2006 and 2008 (Figure 2). The trend in infection did not show any significant association with total rainfall during the preceding year of survey as estimated from meteorological stations in Gezira state (Figure 2).

**Table 1:** Summary of Gezira *P. falciparum *prevalence data from 88 survey locations (968 surveys) from 1999 to 2009 showing annual average infection prevalence, the number of locations with no positive cases, those with ≥5% prevalence and the sample size by residence and year

	% *P. falciparum *positive (number examined)	Survey locations (%) with no positive *P. falciparum *samples	Survey locations (%) with *P. falciparum *prevalence ≥5%
1999	8.6 (8,523)	15 (17.0)	51 (58.0)
2000	4.0 (8,928)	17 (19.3)	28 (31.8)
2001	2.1 (8,667)	39 (44.3)	14 (15.9)
2002	3.7 (8,783)	29 (32.9)	24 (27.3)
2003	2.9 (8,792)	45 (51.1)	14 (15.9)
2004	2.0 (8,763)	32 (36.4)	10 (11.4)
2005	1.9 (8,716)	59 (67.0)	12 (13.6)
2006	2.3 (8,733)	44 (50.0)	16 (18.2)
2007	1.1 (8,794)	49 (55.7)	4 (4.5)
2008	2.1 (8,571)	38 (43.2)	11 (12.5)
2009	1.6 (8,752)	55 (62.5)	10 (11.4)

**Total**	**2.9 (96,022)**		

### Spatial only and space-time clustering in *P. falciparum *infection prevalence

**Figure 3:**
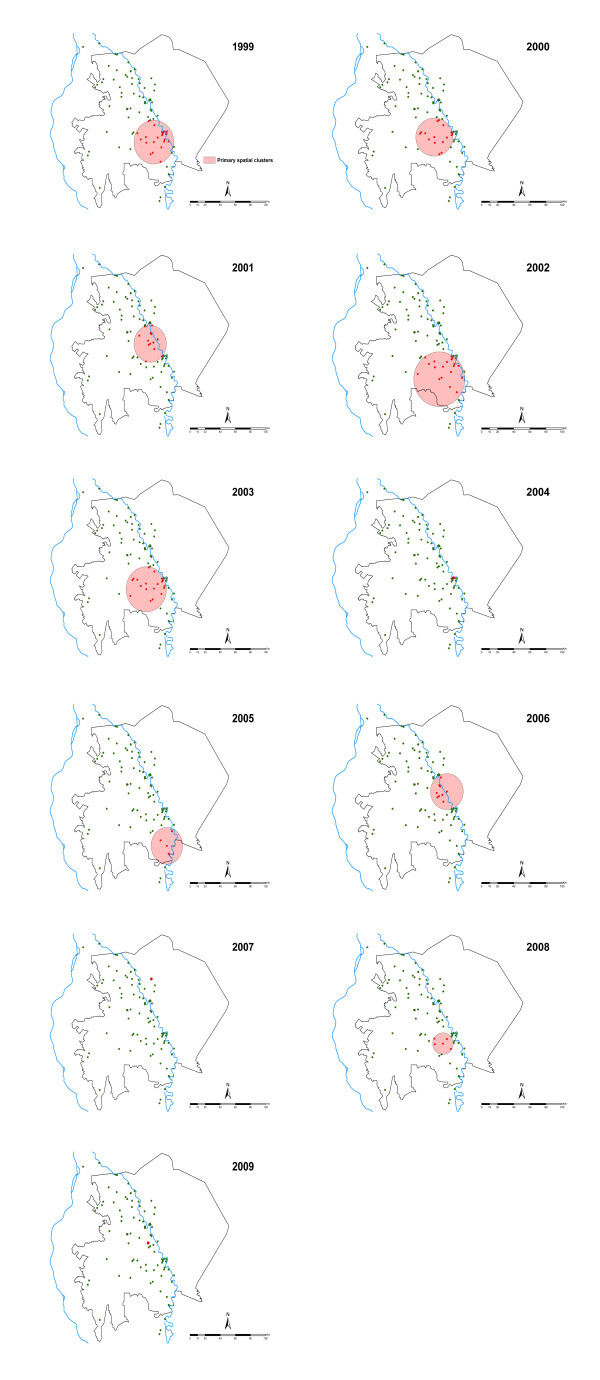
**Location of spatial-only primary clusters of *P. falciparum *prevalence in Gezira state in each year from 1999 to 2009. Primary spatial clusters are shown as pink circles of varying radius or red zeros (when a cluster radius is indeterminate)**. The location of all primary spatial-only clusters, except in 2007 and 2009, appears not vary by large distances and are concentrated in area in south of the Gezira state.

The Kulldorff spatial scan statistic showed highly significant spatial-only clustering of *P. falciparum *prevalence in each year from 1999-2009 (Table 2 and Figure 3). In 2004, 2007 and 2009 the spatial clusters consisted of only one village resulting in clusters of indeterminate radius i.e. only a single village was identified to be in the cluster and hence the resulting cluster radius was 0 km (Table 2). In the other years, the number of villages within a spatial cluster ranged from 26 in 2002 to 6 in 2005 and the radius of the spatial window was from 12.8 km in 2008 to 31.7 km in 2002 (Table 2). In total, 45/88 villages were part of a spatial cluster in one or multiple years over the study period (Figure 3). The rate ratio of the spatial cluster or the risk of an individual within the cluster having an infection compared to those outside was highest in 2009 (RR = 14.66) and lowest in 2001 (RR = 2.20). The proportion of malaria positive cases within a cluster ranged from 8.4% in 2004 to 69.4% in 2005. Across the study period the mean *P. falciparum *prevalence within the spatial-only clusters was between two to 14 times higher than that outside the clusters (Table 2), with the differences generally increasing with year as overall prevalence declined.

**Table 2:** Primary spatial-only *P. falciparum *clusters, their radius, the number of villages and cases contained in the clusters and the significance of the Kulldorff scan statistic in Gezira state from 1999 to 2009

Year	Number of clusters	Number of Villages in cluster	Radius of cluster (Km)	% Examined inside cluster	% *P. falciparum *cases inside cluster	Relative Risk	P-Value	% *P. falciparum *positive inside cluster	% *P. falciparum *positive outside cluster
1999	1	25	26.3	28.1	47.8	2.34	0.0001	14.7	6.3
2000	1	19	23.0	21.3	42.6	2.74	0.0001	8.0	2.9
2001	1	20	21.4	23.0	39.7	2.20	0.0004	3.6	1.6
2002	1	26	31.7	29.8	62.8	3.98	0.0001	7.9	2.0
2003	1	24	26.8	27.2	69.4	6.08	0.0001	7.3	1.2
2004	1	1	0.0	1.1	8.4	7.97	0.0001	15.0	1.9
2005	1	6	20.8	6.9	41.1	9.43	0.0001	69.7	7.4
2006	1	17	20.4	19.2	46.5	3.66	0.0001	86.9	23.7
2007	1	1	0.0	1.1	13.0	13.12	0.0001	14.9	1.1
2008	1	7	12.8	7.9	29.0	4.76	0.0001	40.8	8.6
2009	1	1	0.0	1.1	14.5	14.66	0.0001	16.9	1.2

1999-2009	1	24	26.8	27.3	49.2	2.59	0.0001	5.3	2.0

**Figure 4:**
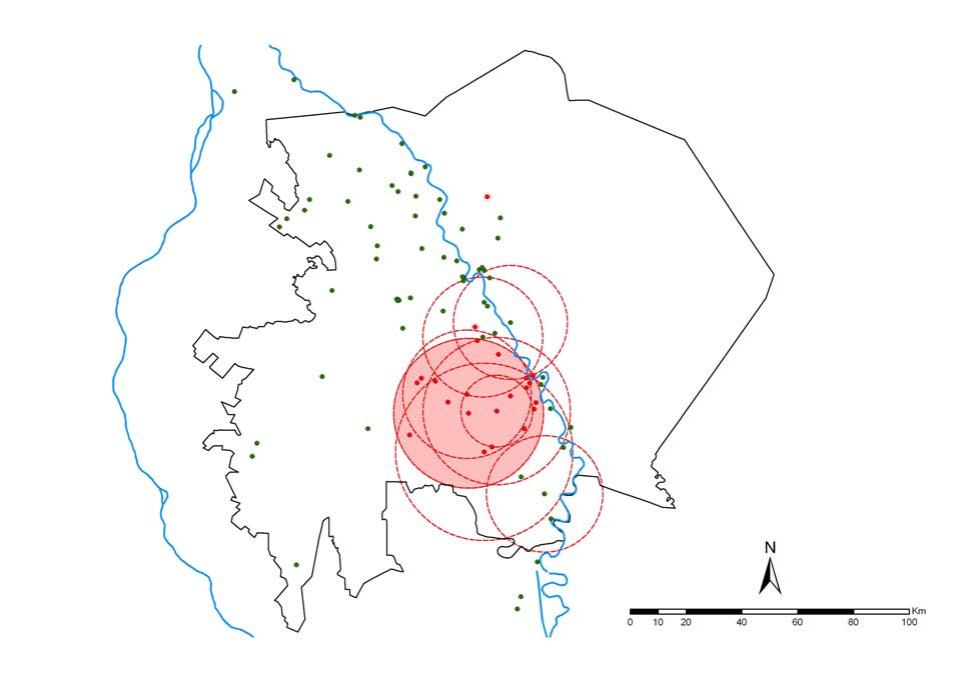
**Location of the space-time primary cluster (Kulldorff statistic was significant at P < 0.01, shaded) of *P. falciparum *prevalence in Gezira state from 1999 to 2009**. Shown also are the spatial only primary clusters for each year (circles with broken red boundaries). Except for the spatial-only cluster in 2007 (northwest of Gezira, on the western side of the Blue Nile), all other spatial-only clusters either overlapped or were contained with the primary space-time cluster.

The Kulldorff space-time scan statistics, however, identified only one highly significant primary space-time cluster of high prevalence centered near the southern tip of Gezira state (Table 2 and Figure 4). This cluster contained 24 villages within a radius of 26.8 km and a rate ratio of 2.59. About 27% of all persons examined and 49% of all those who were positive for *P. falciparum *infection over the study period were contained in this cluster (Table 2). The overall positivity rate inside the space-time cluster was 5.3% compared to 2.0% outside the cluster.

## Discussion

Eleven years of data on *P. falciparum *infection prevalence among children aged two to ten years in 88 villages in Gezira state were assembled. Over this study period, overall infection prevalence reduced from approximately five-fold to just below 2% in 2009 with infection rates dropping to 4% by 2000, the second year of the study, and remaining below this level in all subsequent years (Table 1 and Figure 2). Importantly, using the Kulldorff scan statistics [20,37] it was possible to show the presence of spatial-only and space-time clustering of infection prevalence in Gezira state. In each year, there was at least one significant primary spatial-only cluster containing from one to 26 villages and a single overall single significant primary space-time cluster (Table 2, Figures 3 and 4). The mean prevalence of infection in the villages contained in the primary spatial-only or space-time clusters was consistently higher than that of the overall data or of those villages outside the clusters throughout the study period. All spatial-only clusters either overlapped or were contained with the primary space-time cluster in all the years except in 2007, a year in which the cluster was of indeterminate radius.

The analysis of spatial and temporal clustering of malaria has predominantly been examined in areas of stable or low stable endemic transmission [2,28,29]. This study, however, represents spatial-temporal cluster analysis of malaria infections in a very low unstable transmission area where disease risk manifests as "hotspots" and is associated with occasional epidemics. The results have a number of implications for malaria control in Gezira state. First, the primary space-time cluster identified in this study is located on the southern tip of the state in an area near a sugar plantation close to the Sennar dam which irrigates the very large Gezira scheme. In addition, almost all the spatial-only clusters observed in each year were either within or intersected the primary space-time cluster in the south of Gezira state, indicating a highly focal concentration of infections which can be addressed by focused targeting of interventions. Second, while it is difficult to empirically determine the reason for the peaks in infection prevalence in an otherwise declining trend over the study period using the available data, it is interesting that these coincided with the period when reports of vector resistance to malathion-deltamethrin emerged and its use was discontinued in 2002; when vector resistance to pyrethroid-permethrin had increased to substantial levels in 2006 just before its replacement with bendiocarb in 2007; and in 2008 when the IRS programme was wound up (Figure 2). Therefore, malaria control in Gezira needs to maintain and scale-up its efforts on prevention of disease among the agricultural population in the Gezira irrigation scheme and those along the southern and central Blue Nile River, expand control efforts to the neighbouring Sennar state where the Sennar dam is located.

Although the data assembled for this study provide a useful basis for tracking the changing infection prevalence among the population in Gezira state, there are some caveats and opportunities for strengthening future surveillance. First, the temporal resolution of the data of one survey each year limits the analysis of seasonal peaks of transmission and potentially misses a significant proportion of infections. Second, understanding the microgeographic (households and individual) heterogeneity of malaria infections has potentially important implications for small-area targeting of malaria control. The survey data, however, could not be assembled at the household or individual levels because hardcopy survey data at household and individual level could not be located from the GMCP archives. Future surveys should be digitized to make them amenable to detailed microgeographic analyses that account for potential risk factors such as age, housing structure, behavioural and environmental variables. Third, in the context of elimination and given the low levels of malaria transmission, data on all ages examining not only the asexual stage infections but also the sexual stages are required [38]. Finally, in areas of low malaria transmission microscopy or rapid diagnostic tests have low detection rates [39,40] thereby underestimating the overall infection prevalence. Alternative approaches such as long-term active and passive case-detection from a spatially representative sample of communities and health facilities and use of polymerase chain reaction (PCR) [39] to detect low level infections and serological markers to assess Plasmodium antibody exposure [40,41] should be explored. One of the best examples of such a detailed investigation is represented by the 11 year longitudinal study of malaria in one village, Daraweesh in Eastern Sudan [42], which provided important observations on the epidemiology [43], seasonality [44], presence of sub-microscopic chronic *P. falciparum *infections [45]; ethnic and genetic susceptibility [42,46] and immunity [47] to malaria under conditions of very low transmission intensity.

## Conclusion

This study demonstrates the potential of space-time clustering techniques to identify areas of high malaria infection rates in an area of generally low transmission in Gezira state. The success of malaria elimination in the Gezira, the stated aim of the GMFI, depends critically on sustained control and the establishment of high quality surveillance to measure disease patterns. All of these are linked to the availability of adequate funding but there are already shortfalls in the financing of both control and epidemiological surveillance [33]. The development of better surveillance systems to document any changes in malaria epidemiology of the disease should consider the establishment of health facility, school and community sentinel sites for the prospective assembly of high quality active and passive case detection data.

## Abbreviations

AS+SP: Artesunate+sulphadoxine-pyrimethamine; CQ: chloroquine; DDT: dichlorodiphenyltrichloroethane; GPS: Global Positioning System; GMCP: Gezira Malaria Control Programme; GMFI: Gezira Malaria Free Initiative; LLIN: long-lasting insecticidal nets; ITN: insecticide-treated nets; IRS: indoor residual spraying; NMCP: National Malaria Control Programme; SP: sulphadoxine-pyrimethamine; WHO: World Health Organization

## Competing interests

The authors declare that they have no competing interests.

## Authors' contributions

SEM was responsible for data assembly, cleaning, analysis, interpretation and production of the final manuscript; BYMN was responsible overall supervision of survey data assembly; SMB was responsible overall supervision of survey data assembly; IH was responsible overall supervision of survey data assembly; RWS was responsible for overall scientific management, interpretation and preparation of the final manuscript; AMN was responsible for overall statistical analysis, interpretation and production of the final manuscript. All authors read and approved the final manuscript.

## References

[B1] Cattani JA Tulloch JL Vrbova H Jolley D Gibson FD Moir JS Heywood PF Alpers MP Stevenson A Clancy R The epidemiology of malaria in a population surrounding Madang, Papua New Guinea Am J Trop Med Hyg 1986 35 3 15 3511748 10.4269/ajtmh.1986.35.3

[B2] Gamage-Mendis AC Carter R Mendis C De Zoysa AP Herath PR Mendis KN Clustering of malaria infections within an endemic population: risk of malaria associated with the type of housing construction Am J Trop Med Hyg 1991 45 77 85 1867350 10.4269/ajtmh.1991.45.77

[B3] Greenwood BM The microepidemiology of malaria and its importance to malaria control Trans R Soc Trop Med Hyg 1989 83 25 29 10.1016/0035-9203(89)90599-3 2576161

[B4] Carter R Mendis KN Roberts D Spatial targeting of interventions against malaria Bull World Health Organ 2000 78 1401 11196487 PMC2560653

[B5] Brooker S Clarke S Njagi JK Polack S Mugo B Estambale B Muchiri E Magnussen P Cox J Spatial clustering of malaria and associated risk factors during an epidemic in a highland area of western Kenya Trop Med Int Health 2004 9 757 766 10.1111/j.1365-3156.2004.01272.x 15228485

[B6] Mackinnon MJ Mwangi TW Snow RW Marsh K Williams TN Heritability of malaria in Africa PLoS Med 2005 2 e340 10.1371/journal.pmed.0020340 16259530 PMC1277928

[B7] Coleman M Mabuza AM Kok G Coetzee M Durrheim DN Using the SaTScan method to detect local malaria clusters for guiding malaria control programmes Malar J 2009 8 10.1186/1475-2875-8-68 19374738 PMC2679049

[B8] Stirnadel HA Al-Yaman F Genton B Alpers MB Smith TA Assessment of different sources of variation in the antibody responses to specific malaria antigens in children in Papua New Guinea Int J Epidemiol 2000 29 579 10.1093/ije/29.3.579 10869334

[B9] Trape JF Lefebvrezante E Legros F Ndiaye G Bouganali H Druilhe P Salem G Vector density gradients and the epidemiology of urban malaria in Dakar, Senegal Am J Trop Med Hyg 1992 47 181 189 1354414 10.4269/ajtmh.1992.47.181

[B10] Clarke SE Variation in malaria risk and response in Rural Gambia 2001 University of Copenhagen

[B11] Ernst KC Lindblade KA Koech D Sumba PO Kuwuor DO John CC Wilson ML Environmental, socio-demographic and behavioural determinants of malaria risk in the western Kenyan highlands: a case-control study Trop Med Int Health 2009 14 1258 1265 10.1111/j.1365-3156.2009.02370.x 19772547 PMC3109621

[B12] Pullan RL Bukirwa H Staedke SG Snow RW Brooker S Plasmodium infection and its risk factors in eastern Uganda Malar J 2010 9 2 10.1186/1475-2875-9-2 20044942 PMC2822788

[B13] Lindsay SW Snow RW The trouble with eaves; house entry by vectors of malaria Trans R Soc Trop Med Hyg 1988 82 645 646 10.1016/0035-9203(88)90546-9 3256125

[B14] van der Hoek W Konradens F Dijkstra DS AP H Amerasinghe FP Risk factors for malaria: a microepidemiological study in a village in Sri Lanka Trans R Soc Trop Med Hyg 1998 92 265 10.1016/S0035-9203(98)91003-3 9861392

[B15] Subramanian S Manoharan A Sahu S Jambulingam P Govardhini P Mohapatra SS Das PK Living conditions and occurrence of malaria in a rural community Indian J Malar 1991 28 29 37 1915982

[B16] Killeen GF Smith TA Exploring the contributions of bed nets, cattle, insecticides and excitorepellency to malaria control: a deterministic model of mosquito host-seeking behaviour and mortality Trans R Soc Trop Med Hyg 2007 101 867 880 10.1016/j.trstmh.2007.04.022 17631372 PMC1949412

[B17] Koram KA Bennett S Adiamah JH Greenwood BM Socio-economic risk factors for malaria in a peri-urban area of The Gambia Trans R Soc Trop Med Hyg 1995 89 146 150 10.1016/0035-9203(95)90471-9 7778137

[B18] Smith DL Dushoff J McKenzie FE The risk of a mosquito-borne infection in a heterogeneous environment PLoS Biol 2004 2 e368 10.1371/journal.pbio.0020368 15510228 PMC524252

[B19] Kulldorff M Nagarwalla N Spatial disease clusters: detection and inference Stat Med 1995 14 799 810 10.1002/sim.4780140809 7644860

[B20] Kulldorff M Feuer EJ Miller BA Freedman LS Breast cancer clusters in the northeast United States: a geographic analysis Am J Epidemiol 1997 146 161 9230778 10.1093/oxfordjournals.aje.a009247

[B21] Kulldorff M Heffernan R Hartman J Assuncao R Mostashari F A space-time permutation scan statistic for disease outbreak detection PLoS Med 2005 2 e59 10.1371/journal.pmed.0020059 15719066 PMC548793

[B22] Lawson AB Kulldorff M A review of cluster detection methods 1999 New York: Wiley: Wiley

[B23] Morrison AC Getis A Santiago M Rigau-Perez JG Reiter P Exploratory space-time analysis of reported dengue cases during an outbreak in Florida, Puerto Rico, 1991-1992 Am J Trop Med Hyg 1998 58 287 298 9546405 10.4269/ajtmh.1998.58.287

[B24] Washington CHC Radday JJ Streit TGT Boyd HAH Beach MJM Addiss DGD Lovince RR Lovegrove MCM Lafontant JGJ Lammie PJP Hightower AW Spatial clustering of filarial transmission before and after a Mass Drug Administration in a setting of low infection prevalence Filar J 2004 3 3 3 10.1186/1475-2883-3-3 PMC420477 15128461

[B25] Fe'vre EM Coleman PG Odiit M Magona JW Welburn SC Woolhouse ME The origins of a new *Trypanosoma brucei rhodesiense *sleeping sickness outbreak in eastern Uganda Lancet 2001 358 625 628 10.1016/S0140-6736(01)05778-6 11530149

[B26] Mostashari F Kulldorff M Hartman JJ Miller JR Kulasekera V Dead bird clusters as an early warning system for West Nile virus activity Emerg Infect Dis 2003 9 641 646 12781002 10.3201/eid0906.020794 PMC3000152

[B27] Snow RW Schellenberg JR Peshu N Forster D Newton CR Winstanley PA Mwangi I Waruiru C Warn PA Newbold C Marsh K Periodicity and space-time clustering of severe childhood malaria on the coast of Kenya Trans R Soc Trop Med Hyg 1993 87 386 390 10.1016/0035-9203(93)90007-D 8249058

[B28] Schellenberg JA Newell JN Snow RW Mung'ala V Marsh K Smith PG Hayes RJ An analysis of the geographical distribution of severe malaria in children in Kilifi District, Kenya Int J Epidemiol 1998 27 323 10.1093/ije/27.2.323 9602418

[B29] Gaudart J Poudiougou B Dicko A Ranque S Toure O Sagara I Diallo M Diawara S Ouattara A Diakite M Duombo OK Space-time clustering of childhood malaria at the household level: a dynamic cohort in a Mali village BMC Pub Health 2006 6 286 286 10.1186/1471-2458-6-286 PMC1684261 17118176

[B30] Kreuels B Kobbe R Kreuzberg C Von Reden C Bater K Klug S Busch W Adjei O May J Spatial variation of malaria incidence in young children from a geographically homogeneous area with high endemicity J Infect Dis 2007 197 85 10.1086/524066 18171290

[B31] El Gaddal AA The Blue Nile Health Project: a comprehensive approach to the prevention and control of water-associated diseases in irrigated schemes of the Sudan J Trop Med Hyg 1985 88 2 47 56 4032529

[B32] Petrarca V Nugud AD Ahmed MA Haridi AM Di Deco MA Coluzzi M Cytogenetics of the *Anopheles gambiae *complex in Sudan, with special reference to An. arabiensis: relationships with East and West African populations Med Vet Entomol 2000 14 149 164 10.1046/j.1365-2915.2000.00231.x 10872859

[B33] Federal Ministry of Health Documentation of Khartoum and Gezira Malaria Initiatives Khartoum 2004 1 33

[B34] Haridi AM Inheritance of DDT resistance in species A and B of the *Anopheles gambiae *complex Bull World Health Organ 1972 47 619 4540681 PMC2480826

[B35] Hemingway J Biochemical studies on malathion resistance in *Anopheles arabiensis *from Sudan Trans R Soc Trop Med Hyg 1983 77 477 10.1016/0035-9203(83)90118-9 6636275

[B36] Abdalla H Matambo TS Koekemoer LL Mnzava AP Hunt RH Coetzee M Insecticide susceptibility and vector status of natural populations of *Anopheles arabiensis *from Sudan Trans R Soc Trop Med Hyg 2008 102 263 271 10.1016/j.trstmh.2007.10.008 18054056

[B37] SatScan: Software for the spatial, temporal and space-time scan statistics http://www.satscan.org/

[B38] Distiller GB Little F Barnes KI Nonlinear mixed effects modeling of gametocyte carriage in patients with uncomplicated malaria Malar J 2010 9 60 10.1186/1475-2875-9-60 20187935 PMC2845183

[B39] Okell LC Ghani AC Lyons E Drakeley CJ Submicroscopic infection in Plasmodium falciparum-endemic populations: a systematic review and meta-analysis J Infect Dis 2009 200 1509 1517 10.1086/644781 19848588

[B40] Bousema T Youssef RM Cook J Cox J Alegana V Amran J Noor AM Snow RW Drakeley C Serologic markers for detecting malaria in areas of low endemicity, Somalia, 2008 Emerg Infect Dis 2010 6 393 399 10.3201/eid1603.090732 PMC3322012 20202412

[B41] Drakeley CJ Corran PH Coleman PG Tongren JE McDonald SL Carneiro I Malima R Lusingu J Manjurano A Nkya WM Lemnge MM Cox J Reyburn H Riley EM Estimating medium- and long-term trends in malaria transmission by using serological markers of malaria exposure P Nat Acad Sci USA 2005 102 5108 5113 10.1073/pnas.0408725102 PMC555970 15792998

[B42] Creasey A Giha H Hamad AA El Hassan IM Theander TG Arnot DE Eleven years of malaria surveillance in a Sudanese village highlights unexpected variation in individual disease susceptibility and outbreak severity Parasitol 2004 129 263 271 10.1017/S0031182004005724 15471002

[B43] Giha HA Rosthoj S Dodoo D Hviid L Satti GM Scheike T Arnot DE Theander TG The epidemiology of febrile malaria episodes in an area of unstable and seasonal transmission Trans R Soc Trop Med Hyg 2000 94 645 651 10.1016/S0035-9203(00)90218-9 11198648

[B44] Hamad AA Nugud Ael H Arnot DE Giha HA Abdel-Muhsin AM Satti GM Theander TG Creasey AM Babiker HA Elnaiem DE A marked seasonality of malaria transmission in two rural sites in eastern Sudan Acta Trop 2002 83 71 82 10.1016/S0001-706X(02)00059-1 12062795

[B45] Hamad AA El Hassan IM El Khalifa AA Ahmed GI Abdelrahim SA Theander TG Arnot DE Chronic *Plasmodium falciparum *infections in an area of low intensity malaria transmission in the Sudan Parasitol 2000 120 447 456 10.1017/S0031182099005818 10840974

[B46] Giha HA Nasr A Iriemenam NC Arnot D Troye-Blomberg M Theander TG Berzins K ElGhazali G Pandey JP Antigen-specific influence of GM/KM allotypes on IgG isotypes and association of GM allotypes with susceptibility to *Plasmodium falciparum *malaria Malar J 2009 8 306 10.1186/1475-2875-8-306 20028548 PMC2805690

[B47] Giha HA Nasr A Iriemenam NC Balogun HA Arnot D Theander TG Troye-Blomberg M Berzins K ElGhazali G Age-dependent association between IgG2 and IgG3 subclasses to Pf332-C231 antigen and protection from malaria, and induction of protective antibodies by sub-patent malaria infections, in Daraweesh Vaccine 2010 28 1732 1739 10.1016/j.vaccine.2009.12.018 20036751

